# Landscape of copy number variations in *Bos taurus*: individual – and inter-breed variability

**DOI:** 10.1186/s12864-018-4815-6

**Published:** 2018-05-29

**Authors:** M. Mielczarek, M. Frąszczak, E. Nicolazzi, J. L. Williams, J. Szyda

**Affiliations:** 1 Biostatistics group, Department of Genetics, Wroclaw University of Environmental and Life Sciences, Kozuchowska 7, 51-631 Wroclaw, Poland; 2 National Research Institute of Animal Production, Krakowska 1, 32-083 Balice, Poland; 3 Council on Dairy Cattle Breeding (CDCB), 4201 Northview Dr, Bowie, MD 20716 USA; 4 Davies Research Centre, University of Adelaide, School of Animal and Veterinary Sciences, Roseworthy, SA 5371 Australia

**Keywords:** Copy number variation, Cattle, Genetic diversity, Next-generation sequencing

## Abstract

**Background:**

The number of studies of Copy Number Variation in cattle has increased in recent years. This has been prompted by the increased availability of data on polymorphisms and their relationship with phenotypes. In addition, livestock species are good models for some human phenotypes. In the present study, we described the landscape of CNV driven genetic variation in a large population of 146 individuals representing 13 cattle breeds, using whole genome DNA sequence.

**Results:**

A highly significant variation among all individuals and within each breed was observed in the number of duplications (*P* < 10^−15^) and in the number of deletions (*P* < 10^−15^). We also observed significant differences between breeds for duplication (*P* = 0.01932) and deletion (*P* = 0.01006) counts. The same variation CNV length - inter-individual and inter-breed differences were significant for duplications (*P* < 10^−15^) and deletions (*P* < 10^−15^). Moreover, breed-specific variants were identified, with the largest proportion of breed-specific duplications (9.57%) found for Fleckvieh and breed-specific deletions found for Brown Swiss (5.00%). Such breed-specific CNVs were predominantly located in intragenic regions, however in Simmental, one deletion present in five individuals was found in the coding sequence of a novel gene ENSBTAG00000000688 on chromosome 18. In Brown Swiss, Norwegian Red and Simmental breed-specific deletions were located within KIT and MC1R genes, which are responsible for a coat colour. The functional annotation of coding regions underlying the breed-specific CNVs showed that in Norwegian Red, Guernsey, and Simmental significantly under- and overrepresented GO terms were related to chemical stimulus involved in sensory perception of smell and the KEGG pathways for olfactory transduction. In addition, specifically for the Norwegian Red breed, the dopaminergic synapse KEGG pathway was significantly enriched within deleted parts of the genome.

**Conclusions:**

The CNV landscape in *Bos taurus* genome revealed by this study was highly complex, with inter-breed differences, but also a significant variation within breeds. The former, may explain some of the phenotypic differences among analysed breeds, and the latter contributes to within-breed variation available for selection.

**Electronic supplementary material:**

The online version of this article (10.1186/s12864-018-4815-6) contains supplementary material, which is available to authorized users.

## Background

The analysis of Copy Number Variation (CNV) has been carried out in many species including humans [1, 2], mice [3] and cattle [4, 5]. CNVs are structural polymorphisms, including deletions, insertions and duplications. CNVs in genes and regulatory regions potentially impact phenotypes [6–11] and provide a source of genetic variation. It has been found that CNVs often occur in gene-rich regions and are associated with phenotypic variation as well as disease susceptibility [12, 13]. In livestock, pigmentation, coat colour, body size, olfaction, immune response, pathogen and parasite resistance, lipid and protein metabolism, feed efficiency, fertility and milk production have been found to be affected by CNVs [10, 12, 14, 15].

CNV were originally detected by approaches such as Comparative Genomic Hybridization (CGH), array-based Comparative Genomic Hybridization (aCGH), quantitative Polymerase Chain Reaction (qPCR), or using SNP arrays. So far in cattle CNVs have been detected using SNP array [11, 15–21] while a few studies have used the comparative genomic hybridization approach [22, 23]. However, both methods suffer from low accuracy of CNV location and CNV length estimation, and are not able to detect CNVs along the entire genome sequence. The qPCR method has not been applied on a genome-wide scale and is typically used to explore targeted regions e.g. to validate putative CNVs found using other methods [13]. Recent advances in the next generation sequencing (NGS) technology provide a more accurate approach to identifying not only common, but also rare CNVs, at a base-pair resolution [12]. Studies based on NGS have facilitated the discovery of smaller, previously unknown, CNVs [24]. There have been several studies focusing on CNVs in *Bos taurus* at the population level conducted using NGS [4, 5, 10, 25], however, little is known about their population-wise distribution and their potential impact on phenotypes in cattle. Moreover, the overlap of CNVs detected between studies is very low [5].

In this study, we used a full genome sequence data for 146 individuals representing 13 cattle breeds and merged two algorithms for NGS-based CNV detection. Our goal was to describe the CNV genomic landscape in cattle and assess the degree of within- and between-breed variability in the CVN length and number.

## Results

### The landscape of copy number variation in *Bos taurus*

**Fig. 1:**
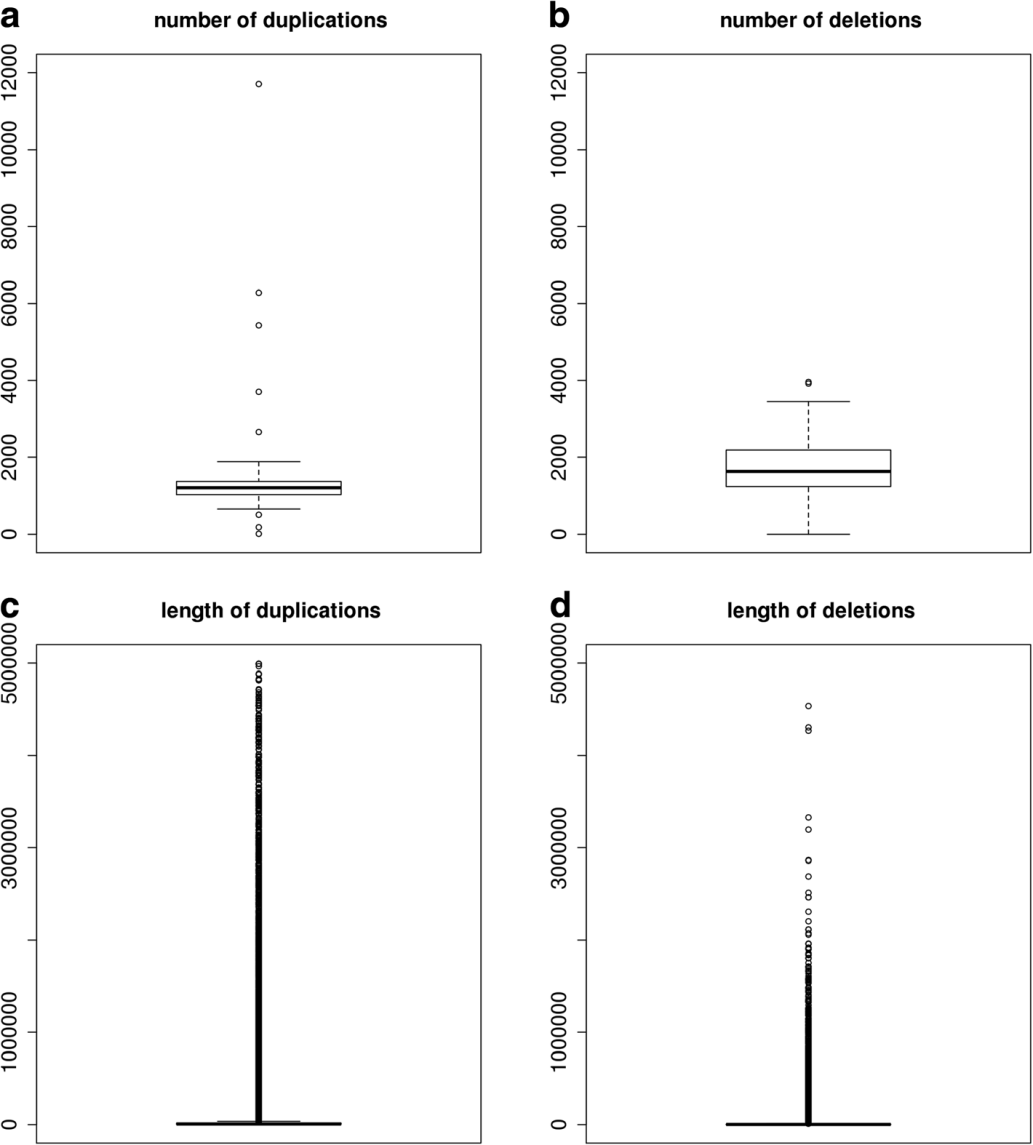
The graphical representation of the number of duplications (**a**) and deletions (**b**) per bull and the length of duplications (**c**) and deletions (**d**) observed in the whole validated data set

The number of CNV variants identified varied considerably among the 146 individuals, ranging between 12 and 11,704 (1343 ± 1086) for duplications and between none and 3960 (1708 ± 700) for deletions. In addition, CNV lengths were also variable, by ranging from 200 bp to 4,992,800 bp (31,018 ± 169,307) for duplications and from 200 bp to 4,536,800 bp (10,836 ± 53,724) for deletions (Fig. 1).

Functional annotation of variants using Sequence Ontology, showed that, 29.49% of duplications and 32.08% of deletions overlapped with genes. The 20 most common duplications, shared by 74–117 bulls representing all breeds and the 20 most common deletions, shared by 117–140 bulls representing all breeds, were examined in detail. Among the most common duplications, there were two duplicated non-protein expressed coding regions. One, located on BTA21, included a transcript of a small nuclear RNA gene (SNORD116, ENSBTAG00000048121) and the other, located on BTA28, was a part of the 5S ribosomal RNA gene (5SrRNA, ENSBTAG00000045518). These two transcripts were classified as having high functional impact. Furthermore, a protein coding region of interferon alpha-inducible protein 27 (ENSBTAG00000003152) on BTA21 was duplicated with potential impact on gene function assigned by the Sequence Ontology. This gene may be involved in regulation of protein export from the nucleus, but it is not well characterized for the cattle genome. A duplication on BTA8 included an intron of the rho-related BTB domain-containing protein 2 gene (RHOBTB2, ENSBTAG00000031916), one duplication on BTA27 included an intron of the enteric beta-defensin gene (EBD, ENSBTAG00000033545), and other two introns were duplicated in the serine/threonine-protein kinase gene (PAK3, ENSBTAG00000015670) on BTX. All of the 14 remaining common duplications were located between genes. The only genic region among the most common deletions occurred in an intron within the uncharacterized gene on BTA6 (ENSBTAG00000035764). The 19 other common deletions were located between genes. However, it is worth mentioning that a partial deletion of MC1R (melanocortin 1 receptor gene) exon. Activation of this gene results in black coat color, whereas loss of function causes red coat color [26]. The deletion was identified in Brown Swiss, Norwegian Red and Simmental individuals, which are red breeds. A list of the 20 most common CNVs, with information on their genomic location and overlaps with polymorphisms from other studies is provided in the Tables 1 and 2.

**Table 1:** The most common duplications in the whole dataset

BTA	begin	end	genomic location	overlapping with the DGVa
2	136,813,001	136,815,100	intergenic	
4	28,200,301	28,203,500	intergenic	
8	70,883,001	70,885,000	intron of the ENSBTAG00000031916 gene (RHOBTB2)	[12] (2)
8	74,685,001	74,687,800	intergenic	
9	53,617,901	53,621,800	intergenic	
18	50,944,801	50,948,100	intergenic	[25] (4), [12] (1), [16] (1)
21	2,128,101	2,130,400	non coding transcript exon of the ENSBTAG00000048121 gene (SNORD116)	
21	59,331,801	59,334,500	coding sequence variant and intron of ENSBTAG00000003152 gene	[12] (1)
27	5,516,501	5,519,500	intron of the ENSBTAG00000033545 gene	[25] (5), [12] (1)
27	28,539,101	28,543,700	intergenic	[25] (2)
27	28,543,901	28,548,300	intergenic	[25] (2)
27	28,548,501	28,552,600	intergenic	[25] (3)
27	28,878,101	28,881,600	intergenic	
28	1,893,701	1,895,100	transcript amplification in the ENSBTAG00000045518 gene (5S rRNA)	[25] (4)
X	36,208,701	36,209,700	intergenic	[25] (1)
X	36,260,901	36,262,400	intergenic	[25] (1)
X	36,673,801	36,676,800	intergenic	[25] (2)
X	64,480,501	64,481,800	intron of the ENSBTAG00000015670 gene (PAK3)	[25] (2), [12] (1)
X	64,504,801	64,512,100	intron of the ENSBTAG00000015670 gene (PAK3)	[12] (1)
X	138,259,801	138,320,600	intergenic	[25] (1)

The list of the 20 most common duplications detected in this study. Genomic locations were determined by the VEP program. The last column shows the number of duplications found in other studies available under the DGVa database

**Table 2:** The most common deletions in the whole dataset

BTA	begin	end	genomic location	overlapping with the DGVa
2	136,815,101	136,816,200	intergenic	[25] (1)
2	136,942,201	136,943,800	intergenic	[25] (1)
6	5,358,201	5,360,200	intergenic	[25] (6)
6	5,897,301	5,899,100	intergenic	[25] (10), [23] (1)
6	5,903,601	5,904,300	intergenic	[25] (10), [23] (1)
6	6,218,501	6,219,600	intron of the ENSBTAG00000035764 gene	[25] (7), [23] (1)
6	6,548,401	6,549,400	intergenic	[25] (8)
7	34,622,901	34,623,700	intergenic	[16] (1), [54] (1)
8	39,388,901	39,389,500	intergenic	[25] (1)
8	62,206,601	62,207,700	intergenic	
14	292,501	294,900	upstream gene variant of ENSBTAG00000046822 (U6 spliceosomal RNA)	[25] (20)
14	322,901	325,800	upstream gene variant of ENSBTAG00000045988 (5S rRNA)	[25] (24)
14	389,001	391,100	downstream gene variant of ENSBTAG00000045780 (5S rRNA)	[25] (26)
16	7,825,301	7,826,200	intergenic	[25] (2)
17	50,668,301	50,670,100	intergenic	[25] (2)
21	2,020,201	2,022,100	upstream gene variant of ENSBTAG00000046925 (5S rRNA)	[25] (1)
21	2,025,201	2,026,700	intergenic	[25] (1)
X	35,728,601	35,730,000	intergenic	
X	53,961,901	53,963,800	intergenic	
X	54,097,401	54,098,700	intergenic	[25] (3)

The list of the 20 most common deletions detected in this study. Genomic locations were determined by the VEP program. The last column shows the number of deletions found in other studies available under the DGVa database

### Inter-individual and inter-breed variation

Most of CNVs, comprising 84.85% of duplications and 77.22% of deletions, were identified in only one bull. There were no identical CNVs, defined as polymorphisms with exactly the same breakpoint positions, which were observed in each of 146 genomes. The most frequent duplication overlapped among 117 bulls and the most common deletion was found in 140 bulls. A highly significant variation among all 146 individuals was observed in the number of duplications and in the number of deletions (both with *P* < 10^−15^). Deletions and duplications were distributed both, within-breeds (with *P* < 10^−15^) and between-breeds (*P* = 0.01932 for duplications and *P* = 0.01006 for deletions). The inter-individual variation in length of CNVs was highly significant for duplications (*P* < 10^−15^) and deletions (*P* < 10^−15^), which was due to both, significant within-breed and between-breed variation. The average length of duplications was highest in Norwegian Red (76,931.9 bp) and lowest in Simmental (13,905.71 bp), which also showed the highest within-breed variation (*P* = 3.02 ∙ 10^−94^). The average length of deletions varied between 7409 bp in Guernsey and 12,564 bp in Fleckvieh and was therefore much lower than for duplications. The highest within-breed variation in deletion length, expressed by *P* = 1.23 ∙ 10^−192^ was found in Norwegian Red. A graphical representation of duplication lengths is provided in Additional file 1: Figure S1 and deletions lengths in Additional file 2: Figure S2. The percentage of the genome containing deletions or duplications among individuals within breeds was significantly different (tests resulting in *p*-values *P* < 0.1 ∙ 10^−12^).

**Fig. 2:**
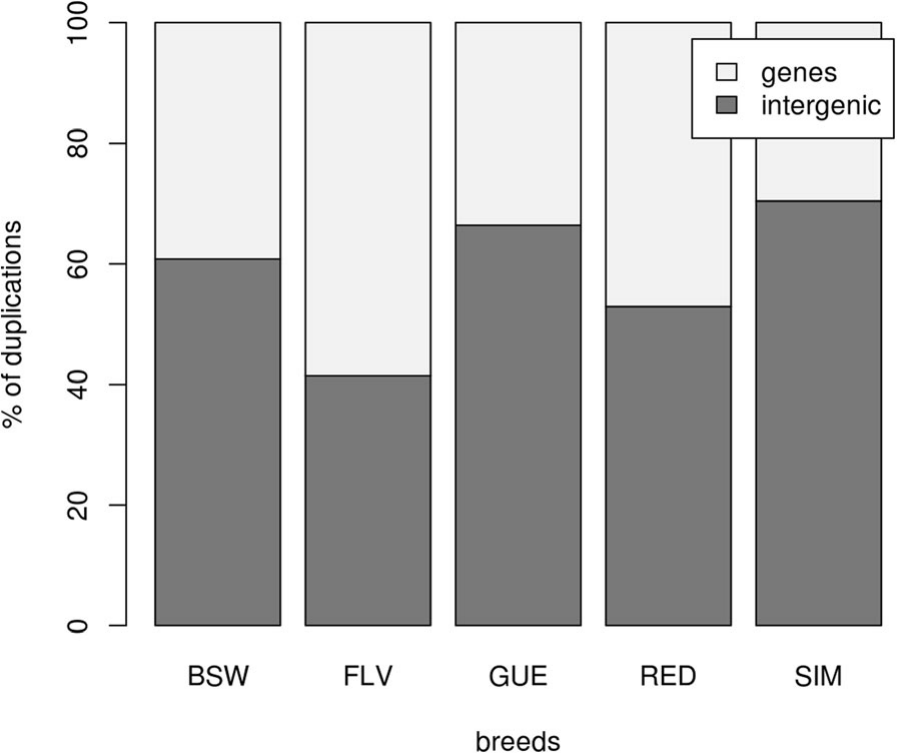
The percent of duplications falling into non-genic and gene regions. BSW represents Brown Swiss, FLV Fleckvieh, GUE Guernsey, RED Norwegian Red and SIM Simmental breed

**Fig. 3:**
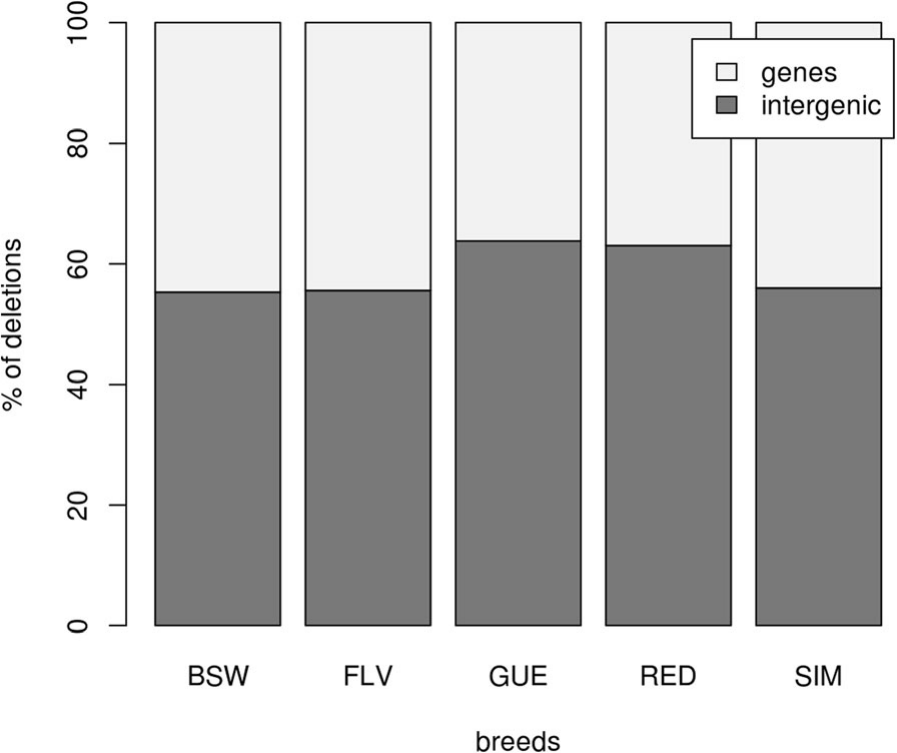
The percent of deletions falling into non-genic and gene regions. BSW represents Brown Swiss, FLV Fleckvieh, GUE Guernsey, RED Norwegian Red and SIM Simmental breed

Functional annotation performed for CNVs separately within each breed showed that the fraction of duplications assigned to gene regions markedly differed between breeds and ranged from 29.56% (Simmental) to 58.61% (Fleckvieh) (Fig. 2). The fraction of deletions ranged from 36.21% (Guernsey) to 44.71% (Brown Swiss) for gene regions (Fig. 3).

### Breed-specific CNVs

**Fig. 4:**
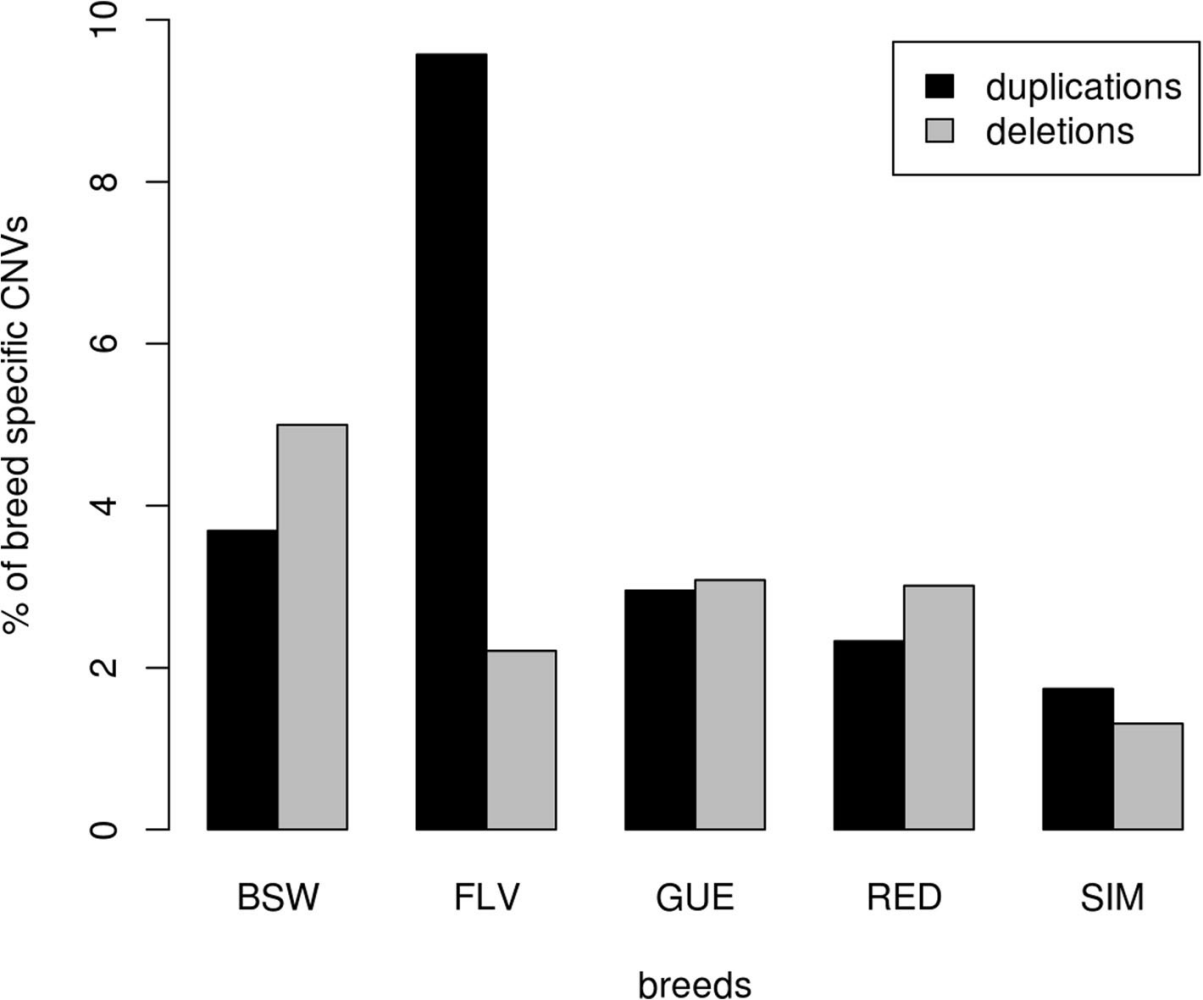
The percent of breed specific deletions/duplications (detected in at least two bulls belonging to the same breed). BSW represents Brown Swiss, FLV Fleckvieh, GUE Guernsey, RED Norwegian Red and SIM Simmental breed

Variants present only in one breed have a potential to contribute to genetic differences between them. Due to still relatively small sizes of breed-specific data sets in this and previous NGS based studies an unequivocal declaration of a CNV being specific for only one breed is not possible. In the present study, breed-specific variants were defined as CNVs shared by at least two bulls within a given breed and absent in the other breeds. The percent of breed-specific CNVs was the lowest in Simmental (1.74% of duplications and 1.31% for deletions), while the most distinct breeds were Brown Swiss with 5.00% of the breed specific duplications while Fleckvieh had 9.57% of the breed specific deletions (Fig. 4). Interestingly, we found that the part of the KIT (the Hardy-Zuckerman 4 feline sarcoma viral oncogene homolog) gene, which explains a considerable proportion of the variation in pigmentation pattern [27], was deleted in five Brown Swiss individuals and was present in all four remaining breeds which have a characteristic spotted phenotype.

Functional annotation of breed-specific duplications showed that the same GO term “detection of chemical stimulus involved in sensory perception of smell” (GO:0050911) was significantly underrepresented in Norwegian Red and significantly overrepresented in Guernsey and Simmental breeds. For Fleckvieh, neither biological process, nor molecular function was significantly under- or overrepresented GO terms found. The overall count of significantly underrepresented and overrepresented GO terms was highest in Simmental. Underrepresented GO terms were mainly related to cell management (e.g. organelle organization, cell differentiation, cellular response to organic substance, regulation of cell proliferation) while overrepresented GO terms were mainly related to immune response (e.g. immunoglobulin production, autophagy and antigen processing and presentation of peptide antigen via MHC class I). Norwegian Red was the breed in which the breed-specific deletions were most significantly underrepresented (e.g. natural killer cell mediated cytotoxicity, immunoglobulin production) and overrepresented (e.g. small molecule metabolic process, response to cytokine, RNA processing, translation) GO terms. A common feature of breed-specific deletions was their significant overrepresentation in the “natural killer cell lectin-like receptor binding” ontology (GO:0046703). In the context of KEGG pathways, the olfactory transduction pathway (bta04740) was significantly enriched among duplicated genes in Guernsey (*P* = 7.80 ∙ 10^−5^) and Simmental (*P* = 7.01 ∙ 10^−22^), while the same pathway (bta04740, *P* = 0.0063) together with dopaminergic synapse (bta04728, *P* = 0.03674) pathway were significantly enriched among deleted genes in Norwegian Red breed.

The most common breed-specific duplications were shared by ten bulls in Brown Swiss (20.83%), seven bulls in Norwegian Red (36.84%), six bulls in Fleckvieh (20.00%), six bulls in Guernsey (30.00%) and five in Simmental (31.25%). The most common breed-specific deletions were present in 23 individuals of the Brown Swiss breed (47.92%), 11 Norwegian Red individuals (57.90%), ten Guernsey (50.00%) and five Simmentals (31.25%).

The genomic annotation of the ten most common duplications and eight deletions within each breed were investigated further. Seven duplications were in intergenic regions and three duplications were located in introns or upstream gene regions (Table 3). In the case of the deletions, five were annotated in intergenic regions, two in introns or upstream gene regions and only one overlapped with a coding sequence. The latter was on the BTA18 and incorporated the exonic sequence of the ENSBTAG00000000688 gene, in which protein product is not well characterized in a mammal genome (Table 4). This gene has been reported to be involved in the regulation of transcription in humans by [28]. The deletion identified in the present study, spanning this gene region was found in five bulls belonging to the Simmental breed, and it is also present in the Database of Genomic Variants [12].

**Table 3:** The most common breed specific duplications

breed	# bulls sharing duplication	BTA	begin	end	genomic location	overlapping with the DGVa
BSW	10	5	74,078,801	74,086,100	intergenic	
BSW	10	14	64,001	89,100	intergenic	[25] (1), [12] (3), [23] (1)
FLV	6	17	72,899,301	72,924,700	intron of the ENSBTAG00000031160 gene	[25] (1), [12] (3), [23] (1), [54] (1)
GUE	6	5	114,221,601	114,225,800	intergenic	
GUE	6	8	56,717,001	56,730,400	intergenic	
GUE	6	12	73,428,801	73,437,300	intergenic	[25] (28)
GUE	6	25	19,009,101	19,013,400	intron of the ENSBTAG00000018560 (DNAH3) gene	
RED	7	9	88,596,301	88,599,700	intron of the ENSBTAG00000015935 (IYD) gene	[25] (2), [12] (1)
RED	7	X	36,034,701	36,036,900	intergenic	[25] (1)
SIM	5	10	24,513,701	24,528,400	intergenic	[25] (14), [12] (1)

The list of the most common duplications detected within each breed. Genomic locations were determined by the VEP program. The last column shows the number of duplications found in other studies available under the DGVa database

**Table 4:** The most common breed specific deletions

breed	# bulls sharing deletion	BTA	begin	end	genomic location	overlapping with the DGVa
BSW	23	5	23,616,701	23,623,400	intergenic	
FLV	5	12	76,499,501	76,514,300	intergenic	[25] (11)
FLV	5	16	23,946,401	23,947,000	intergenic	
FLV	5	18	63,804,801	63,808,200	upstream gene variant of ENSBTAG00000000688	
FLV	5	28	7,026,301	7,027,000	intron of the ENSBTAG00000020361 (SLC35F3) gene	
GUE	10	2	55,348,801	55,371,300	intergenic	
RED	11	17	25,081,301	25,083,200	intergenic	
SIM	5	18	63,800,101	63,806,400	start lost, coding sequence, 5’ UTR, intron of the ENSBTAG00000000688 gene	[12] (1)

The list of the most common deletions detected within each breed. Genomic locations were determined by the VEP program. The last column shows the number of deletions found in other studies available under the DGVa database

The most common breed-specific CNVs overlapping with QTL represented six phenotypic groups: reproduction, milk, production, exterior, meat and carcass as well as health. In the case of duplications, QTL falling into meat and carcass trait class were found in all breeds, except Norwegian Red. For the latter breed duplications occurred in only two QTL, for calving index and length of productive life. Fleckvieh and Simmental specific duplications overlapped with QTL related to milk yield. Interestingly, Simmental specific duplication fell into all phenotypic groups, but deletion overlapped only with body weight. Breed specific deletions in QTL related to body weight were found in all analysed breeds. Breed-specific deletions were also found in QTL for milk yield as well as meat and carcass classes in all breeds except the Simmental breed.

## Discussion

The present study investigated the occurrence of CNVs in 13 breeds of domestic cattle, focussing on inter-individual and inter-breed levels of variation in length, number and function of the variants.

### CNV dataset

Although algorithms for CNV detection have improved recently and are based on improved data provided by the next generation sequencing (NGS), the number of false positive CNV calls are still high [29, 30]. The problem with reliable detection of CNVs has been discussed by [31], who compared CNVs detected for the same individual using three different methods (NGS, oligonucleotide array, CGH array). They observed that there was only a 23% overlap in the CNVs detected. Other authors have also observed a low correlation among CNV detected within and among studies [5, 10] which is caused by technical aspects such as different sample sizes, differences in breeds studied, detection platforms used (array-based vs. NGS) and CNV detection algorithms.

Because of this low reproducibility in CNV detection, it is important that data is carefully edited and results validated. In the present study the raw output was rigorously edited by discarding CVN variants outside the length range 50 bp - 5,000,000 bp. CNVs longer than five Mbp were classified as artefacts of the alignment process. The validated dataset retained only 30.28% of duplications and 11.50% of deletions initially identified in the raw output. It is worth noting that 44% of duplications and deletions detected in the present study fell within or overlapped with CNVs present in the DGVa (https://www.ebi.ac.uk/dgva) and therefore can be considered as validated.

### Genomic landscape of CNVs

The total number of putative CNVs identified in this study was 445,791 (196,241 duplications and 249,550 deletions) with, on average, 3053 CNVs (1344 duplications, 1709 deletions) per bull. In contrast, [31] reported 520 CNVs for one bull, while [32] 790 CNVs for two animals. Furthermore, [10] detected 6811 deletions for 32 animals, while [25] only 547 deletions and 410 duplications for 62 bulls. The number of CNVs in this study was higher which may be explained by the bigger sample size and that most of CNVs were specific for only one animal. Most studies report that deletions are more common than duplications. A possible biological explanation for this is that a non-allelic homologous recombination, one of the major sources of CNVs, generates more deleted than duplicated regions [33]. In the present study, the excess of deletions may also be explained by the CNV detection algorithm used, which applies more stringent criteria for calling duplications, as these are susceptible to the systematic read mapping bias caused by unknown regions in the reference genome [34]. The length of CNVs reported in different studies also differs considerably. In our study, the minimum reported CNV length was constrained by the 200 bp, cut of set in the software. The largest CNVs reported are much longer than CNVs reported by other authors: a maximum CNV length 28 kbp in [32] and 129,9 kbp in [31] in comparison with 4993 kbp for duplications and 4537 kbp for deletions reported in this study. These differences are probably a result of the different CNV detection software and validation methods used. Previous results have reported that CNVs comprise between 1.74 to 10% of the bovine genome [10–12, 25].

### Functional annotation

CNVs often include functional elements of the genome, such as genes or regulatory sequences, and thus have a potential to affect phenotypes [6–11]. In the present study, 29% of duplications and 32% of deletions were assigned to SO terms corresponding to gene regions. However, among the 20 most common deletions only one was located within an intronic part of a gene. Whereas seven of the 20 most common duplications were in two non-protein coding expressed regions, one was within a protein coding region and four were within introns. This suggests that deletion events in coding regions are less evolutionary accepted than duplications. Deletions may have a greater impact on phenotype by interrupting gene products and causing loss of their biological functions [8].

### Inter-individual and inter-breed variation

In this study, a highly significant variation was observed both in the number and length of duplications and in the number and length of deletions among the 146 animals. An inter-individual, breed-independent component was identified. However, most of the CNVs, comprising 84.85% of duplications and 77.22% of deletions, were found in only one bull. A similar proportion was also observed by [25], where 61% of all CNVs were specific to only one animal. CNVs, with exactly the same breakpoints among all 146 individuals, were not observed in our dataset. Considering CNVs which are common to all individuals, it is important to bear in mind that such CNVs might be an artefact arising from the animal used to create the reference bovine genome [25], or artefacts resulting from assembly problems [9]. The proportion of CNVs located in gene regions differed between breeds. Although, as expected, most of CNVs were located in non-genic regions, for the Fleckvieh breed the percent of duplications was higher in genes than in non-genic regions. Fleckvieh also differed from other breeds in as much as it contained a higher proportion of breed-specific duplications. Those duplications seem to reflect the selection history. Since a large number of duplications, especially duplications of coding sequences, enhances organism genetic diversity by allowing to gain new function by duplicated genes [34]. Such diversity may have been promoted for Fleckvieh as it has always been selected as a dual purpose breed. Also [35] observed a high haplotype diversity of Fleckvieh as compared to Simmental, Brown Swiss and Spanish cattle. Moreover, the diversity is reflected by a large effective population size estimated by [36] and being approximately 3 times higher than for the Holstein breed.

It is widely known that CNV type polymorphisms may cause differences in the coat color in cattle [26, 27, 37]. In this study we observed that the part of the KIT (the Hardy-Zuckerman 4 feline sarcoma viral oncogene homolog) gene which explains a considerable proportion of the variation in patterned pigmentation [27] was deleted in Brown Swiss and was present in four remaining breeds having a characteristic spotting phenotype. Contrarily, [37] observed a duplication nearby segment of the KIT gene resulting of serial translocation leading to differential skin color pigmentation in Brown Swiss animals. This particular duplication located on BTA6 was not found in this study for Brown Swiss population. However, we observed an overlapping duplication in one Simmental genome. This founding also overlapped with the CNV gain detected by [5] where bulls representing the seven most popular breeds in the United States (including Simmental) were investigated. On the other hand, following [37] study we also observed the duplication on BTA29 in one Brown Swiss genome which were reported in the context of color sidedness in cattle. What’s interesting, we also detected that MC1R (melanocortin 1 receptor), whose permanent activation results in black coat colour, whereas loss of function mutations causes red coat colour in different cattle breeds [26], was partly deleted in Brown Swiss, Norwegian Red and Simmental individuals.

Although many breed-specific GO terms and KEGG pathways were identified, we have no recognized any systematic pattern of inter-breed differences. Nevertheless, olfactory receptors genes were reported to be duplicated within the bovine genome suggesting that they may be under strong selection for newly evolving functions [26]. This was confirmed here by significantly under – and overrepresented GO terms related to chemical stimulus involved in sensory perception of smell in Guernsey, and Simmental Norwegian Red breeds.

## Conclusions

Structural genomic variations, especially long deletions and duplications, are a common feature in the bovine genome. Compared to SNPs and indels, CNVs show a greater inter-individual variability. In the present study a large proportion of the variants identified were individual specific and are likely to contribute to phenotypic differences between individuals. The diversity of the olfactory gene family, where several CNVs were identified, reveals the possible role of these structural variants in driving functional evolution. While the impact of point mutations, which are predominantly located in gene promoters acts in regulation of expression levels [38], the impact of structural duplications may be in the formation of new genes [39]. Also in the present study we observed that common duplications were more often located in genic regions than common deletions.

## Methods

### Material

Whole genome DNA sequences were generated as described in [40]. In brief: DNA was isolated from blood samples of 155 bulls using a DNA Isolation System, then libraries were generated from 1 μg of genomic DNA using the Illumina TruseqDNA PCR, and sequenced on the IlluminaHiSeq2000 with a 100 cycles of paired-end sequencing module using the Truseq SBS kit v3. All animals were selected and sequenced within the frame of the Gene2Farm project and represented 13 breeds: Brown Swiss (48), Fleckvieh (31), Norwegian Red (26), Guernsey (20), Simmental (16), Parda de la Montaña (4), Pezzata Rossa Italiana (3), Avileña (2), Bruna Italiana (1), Albera (1), Rubia Gallega (1), Toro de Lidia (1) and Pirenaica (1). The total number of raw reads obtained for a single bull varied between 83,423,880 (a Norwegian Red bull) and 763,594,929 (a Brown Swiss bull). The number of reads per individual was shown on Additional file 3: Figure S3 The length of single read was 101 bp and the corresponding insert size was 350 bp. Data were paired-end type and the average quality of reads per bull ranged from 28.11 to 36.69.

### CNV detection and annotation pipeline

**Fig. 5:**
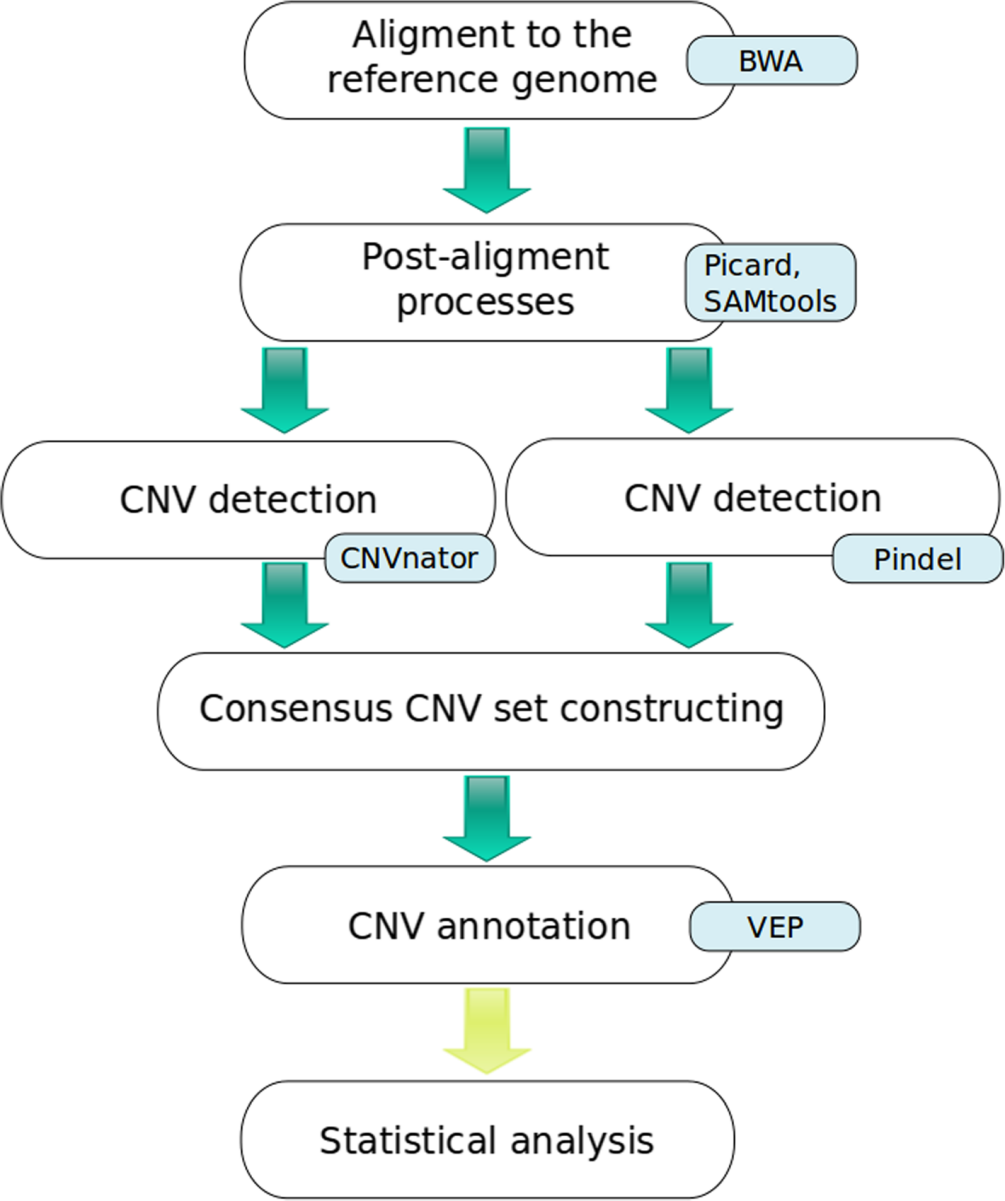
The workflow including CNV detection and annotation pipeline used in this study. White boxes represent particular processes names, while blue boxes represent software. Consensus CNV set constructing and statistical analysis were implemented in self-written scripts

**Fig. 6:**
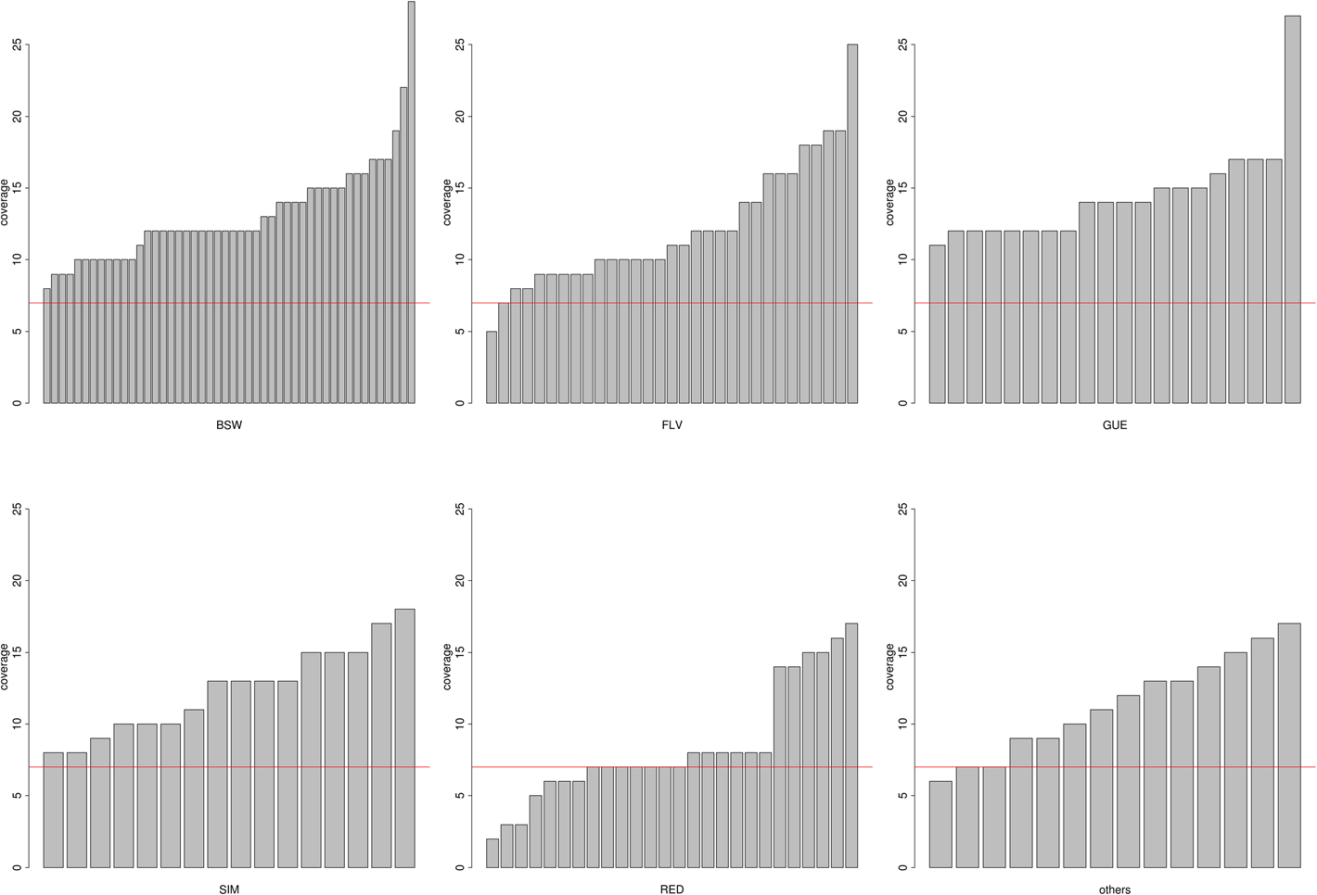
The average genome coverage per individual. Bulls excluded from further analysis are below the red horizontal line. BSW represents Brown Swiss, FLV Fleckvieh, GUE Guernsey, RED Norwegian Red and SIM Simmental breed. The “other” category contains individuals belonging to breeds such as Parda de la Montaña (4 bulls), Pezzata Rossa Italiana (3), Avileña (2), Bruna Italiana (1), Albera (1), Rubia Gallega (1), Toro de Lidia (1) and Pirenaica (1)

Annotated CNVs were performed using the following steps, described in detail below: (i) an alignment to the reference bovine genome, (ii) data processing after alignment, (iii) CNV detection, (iv) validation of CNVs, and (v) CNVs annotation (Fig. 5). BWA-MEM software [41] was used to align reads against the UMD 3.1 [42] reference bovine genome. Post alignment processing was done using a collection of tools from the Picard (http://broadinstitute.github.io/picard/) and the SAMtools packages [43]. This step included converting a SAM format to a BAM format, merging BAM files, sorting reads, removing identical duplicates, and sequence indexing. The average coverage per individual was calculated by using the following formula:

1 $$ coverage=\frac{\sum_{i=1}^N{r}_i}{d}, $$

where *N* denoted the total number of aligned reads, *r*_*i*_ was length in bp of i-th read and *d* the length of the reference genome (2697.56 Mb). This value was used to exclude individuals with an average genome coverage below seven from downstream analyses. As a consequence, nine individuals (seven Norwegian Red, one Fleckvieh and one Parda de la Montaña) were discarded (Fig. 6). In order to control the alignment process (i) the percent of all aligned reads and (ii) the percent of properly paired reads (aligned to the same chromosome with the reasonable insert size and oriented towards each other) were determined. Because the percent of all aligned reads was fairly high (86.87% in one Brown Swiss bull and from 96.01 to 99.92% for the others) as well as the percent of properly paired reads (from 80.62 to 99.14%.) we did not exclude any other animal from the analysis. Therefore, the CNV detection was carried out for 146 bulls. CNV were detected with the CNVnator [44] and the Pindel [45] programs. The read-depth (RD) algorithm implemented in the CNVnator software is based on the comparison of genome coverage and assumes that regions with coverage different from the genome average correspond to CNVs [46]. The Pindel program is based on the split-read (SR) approach, which uses paired-end reads features for CNV detection. CNVs detected by the CNVnator software, longer than 5,000,000 bp were discarded as were CNVs detected by Pindel which were outside the length range of 50 bp - 5,000,000 bp. The consensus set was then created, using the output of the CNVnator as a baseline data set and each variant, which was also detected by the Pindel software was classified as validated. This validated dataset was compared to CNVs available in the Database of Genomic Variants archive (DGVa). Only CNVs classified as the gain (duplications) or loss (deletions) of DNA fragment, which is consistent with the CNVnator output, were used and other variants available in the database e.g. assigned as “inversions” were excluded. The breakpoint position accuracy implemented in CNVnator was 100 bp, therefore, for all comparisons, breakpoint positions within the range 100 bp up- or downstream, were considered as the same. CNVs were annotated using the Variant Effect Predictor software [47] and classified as genic or non-genic (defined as described in the Additional file 4: Table S1). Predicted consequences of deletions or duplications were assigned according to the Sequence Ontology (SO) classification [48] for the 20 most common duplications and the 20 most common deletions identified in the whole dataset, as well as for the most common breed-specific duplications and deletions (shared by at least two individuals within a breed). Breed specific CNVs were subjected to enrichment analysis of underlying GO terms [49, 50] and KEGG pathways using the Kobas software [51, 52]. The most common breed specific CNVs were also compared with QTL from the AnimalQTLdb (www.animalgenome.org/). Breed specific CNVs were analysed for the five most numerous breeds: Brown Swiss, Guernsey, Fleckvieh, Simmental and Norwegian Red.

### Testing inter-individual and inter-breed variation in CNVs

Inter-individual and the inter-breed variation in the number of variants was tested separately for duplications and deletions using:

2 $$ {\chi}^2=\sum \limits_{i=1}^m\frac{{\left({O}_i-E\right)}^2}{E}\sim {\chi}_{m-1}^2, $$

where *O*_*i*_ denotes the number of duplications/deletions for *i*-th individual, *E* is the average number of deletions/duplications identified in the whole dataset and *m* denotes the number of bulls. For Brown Swiss, Guernsey, Fleckvieh, Simmental and Norwegian Red the *χ*^2^ test was used within-breed, where E represents a breed-specific average number of deletions/duplications.

In order to test the variability in the number of deletions/duplications among breeds the Kurskal-Wallis test was performed:

3 $$ H=\frac{12}{n\left(n+1\right)}\sum \limits_{i=1}^m\frac{R_i^2}{k_i}-3\left(m+1\right)\sim {\chi}_{n-1}^2 $$

where *k*_i_ is the number of individuals representing *i*-th breed, and $n=\sum \limits_{i=1}^m{k}_i$, *m* is the number of breeds and *R*_*i*_ denotes the sum of ranks of the deletion/duplication count in *i*-th breed.

The null hypothesis that lengths of deletions/duplications are normally distributed was tested using the Shapiro-Wilk test:

4 $$ W=\frac{{\left[{\sum}_{i=1}^{\left[\frac{n}{2}\right]}{a}_i(n)\left({X}_{\left(n-i+1\right):n}-{X}_{i:n}\right)\right]}^2}{\sum_{i=1}^n{\left({X}_i-\overline{X}\right)}^2}, $$

where *a*_*i*_ represents a constant from Shapiro-Wilk tables, *n* denotes the number of CNVs, *X*_*i* : *n*_ is the length of *i*-th variant in the sorted vector of variants length.

As CNV lengths did not follow a normal distribution, in order to test whether the distribution of CNV lengths is the same for all individuals a Kruskal–Wallis test was applied as in equation (3) but variables were denoted as follows: *k*_i_ was the number of duplications/deletions for *i*-th bull, and $k=\sum \limits_{i=1}^n{k}_i$, *m* was the number of bulls and *R*_*i*_ denoted the sum of ranks for deletion/duplication length corresponding to *i*-th bull. The same test was applied to check whether variability in the length of deletions/duplications between breeds exists.

The difference in the percentage of genome covered by CNVs was tested between individuals within-breed with the null hypothesis that for each bull the same percentage of the genome is covered by deletions /duplications. The hypothesis was tested using the multiple proportion test:

5 $$ F=\frac{\sum_{i=1}^ld\bullet {\left({p}_i-\overline{p}\right)}^2}{\sum_{i=1}^l{p}_i\bullet \left(1-{p}_i\right)}\bullet \frac{l}{l-1}, $$

where *p*_*i*_ denotes the observed percentage of the genome of the *i*-th individual covered by CNVs, $\overline{p}$ denotes the mean of *p*_*i*_, *d* is the length of the reference genome, and *l* is the number of animals representing a given breed. Under the null hypothesis, this test statistic follows the F(l − 1, t) distribution, where *t* → ∞. Nominal *P*-values for each breed were subjected to Bonferroni correction for multiple testing. The statistical analysis was performed in R package [53]. Inter-individual within-breed variation and inter-breed variation was tested for the Brown Swiss, Guernsey, Fleckvieh, Simmental and Norwegian Red breeds.

## Additional files

Additional file 1: **Figure S1.** The length of duplications found within each breed. BSW represents Brown Swiss, FLV Fleckvieh, GUE Guernsey, RED Norwegian Red and SIM Simmental breed. (TIFF 22406 kb)

Additional file 2: **Figure S2.** The length of deletions found within each breed. BSW represents Brown Swiss, FLV Fleckvieh, GUE Guernsey, RED Norwegian Red and SIM Simmental breed. (TIFF 22494 kb)

Additional file 3: **Figure S3.** The number of reads per individual (in millions). (TIFF 21489 kb)

Additional file 4: **Table S1.** SO terms classified in two, more general groups as the non-genic and genic regions. (XLSX 8 kb)

## Abbreviations

aCGH: Array-based comparative genomic hybridization
BAM: Binary version of a SAM file
BTA: *Bos taurus* autosome
BTX: *Bos taurus* X chromosome
BWA: Burrows-Wheeler Aligner
CGH: Comparative genomic hybridization
CNV: Copy number variation
NGS: Next generation sequencing
PCR: Polymerase chain reaction
qPCR: Quantitative polymerase chain reaction
RD: Read-depth
SAM: Sequence alignment/map file format
SNP: Single nucleotide polymorphism
SR: Split-read
VEP: Variant effect predictor
