# Supplementary figures and images for: Landscape of copy number variations in Bos taurus: individual – and inter-breed variability
Source: BMC Genomics. 2018 May 29;19:410. doi: 10.1186/s12864-018-4815-6 (PMC5975385; doi:10.1186/s12864-018-4815-6)

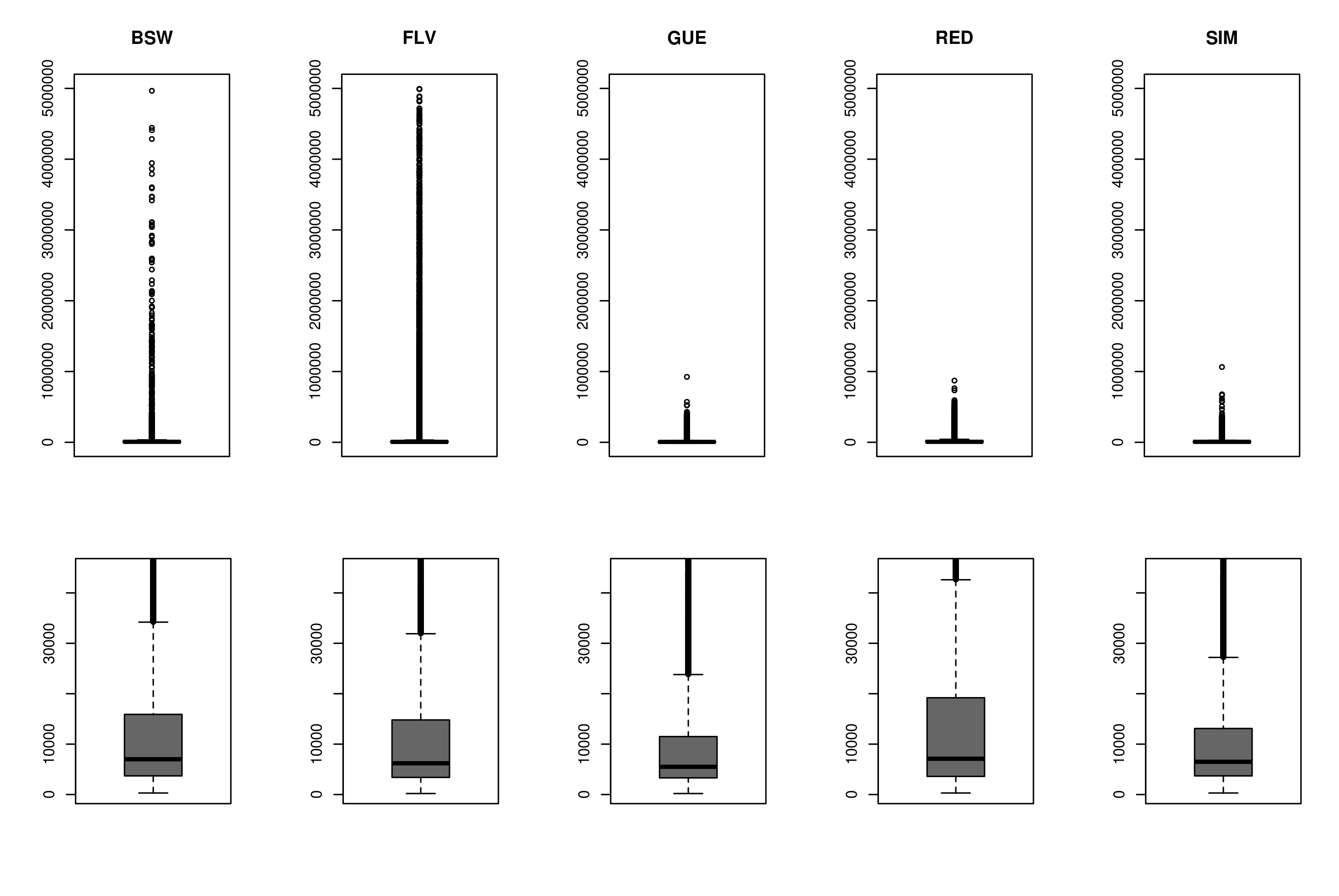

Supplement: Supplementary file 1 — Figure S1. The length of duplications found within each breed. BSW represents Brown Swiss, FLV Fleckvieh, GUE Guernsey, RED Norwegian Red and SIM Simmental breed. (TIFF 22406 kb) [file 12864_2018_4815_MOESM1_ESM.tiff]

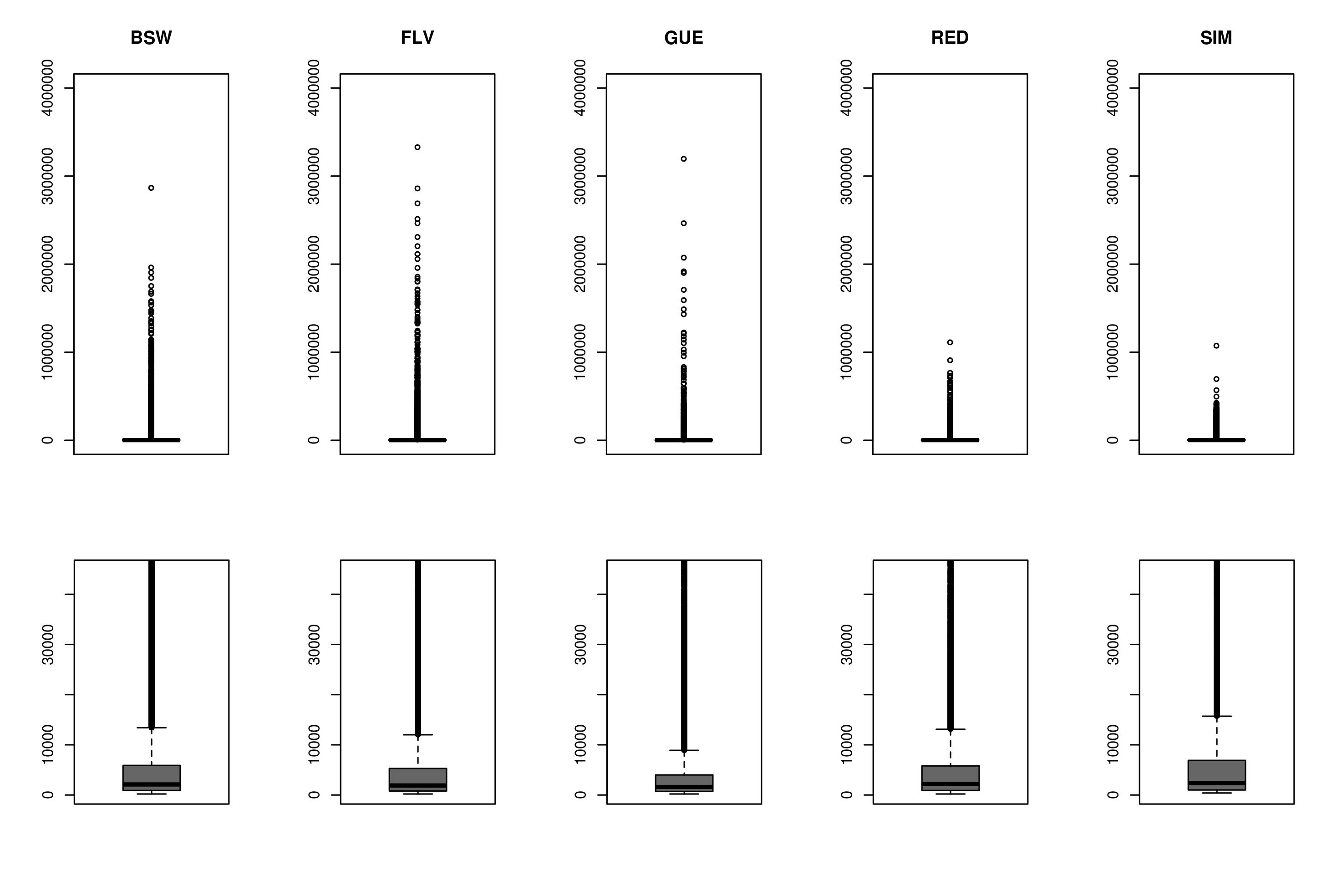

Supplement: Supplementary file 2 — Figure S2. The length of deletions found within each breed. BSW represents Brown Swiss, FLV Fleckvieh, GUE Guernsey, RED Norwegian Red and SIM Simmental breed. (TIFF 22494 kb) [file 12864_2018_4815_MOESM2_ESM.tiff]

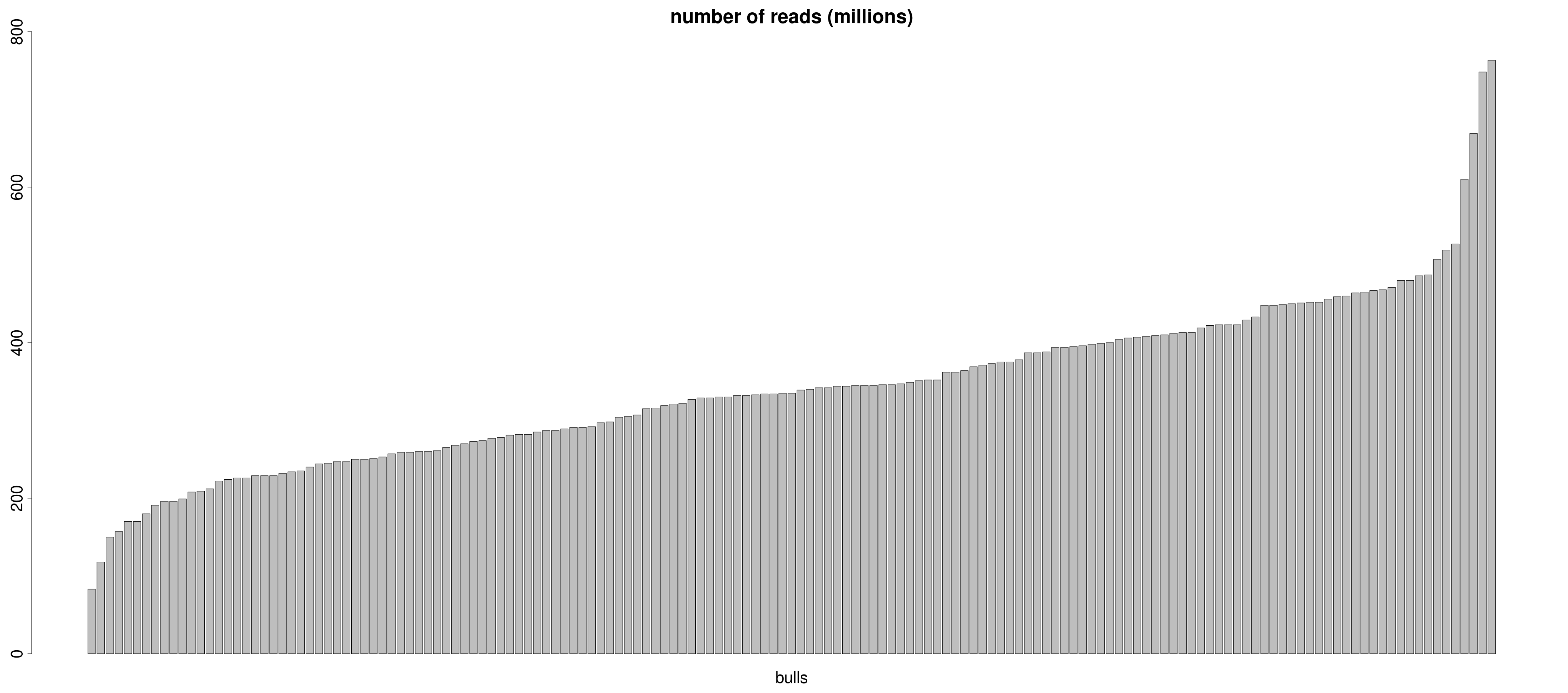

Supplement: Supplementary file 3 — Figure S3. The number of reads per individual (in millions). (TIFF 21489 kb) [file 12864_2018_4815_MOESM3_ESM.tiff]
